# Supplementary material for: Protective Efficacy of Anti-Hyr1p Monoclonal Antibody against Systemic Candidiasis Due to Multi-Drug-Resistant Candida auris
Source: J Fungi (Basel). 2023 Jan 12;9(1):103. doi: 10.3390/jof9010103 (PMC9860579; doi:10.3390/jof9010103)
Supplement: Supplementary file 1 [file jof-09-00103-s001.zip › jof-2142741-supplementary.pdf]

A. Mouse hybridoma clones produced anti-Cal-Hyr1#peptide5 monoclonal antibodies

**Clone ID.**	*6F7A11*	*8G9D5 (HX01)*	*6F7C6 (HX02)*	*8G9D9*	*4A6C6*	*4A6H2*	*3G4E1*
**OD450**	2.158	1.699	1.726	1.745	1.426	1.387	1.992
**Clone ID.**	*3G4H8*	*2B3B4*	*2B3B9*	*7G9F12*		*POS*	*NEG*
**OD450**	1.941	1.975	2.027	1.970		2.169	0.098

B. Monoclonal anti-Cal-Hyr1#peptide5 MAbs recognize native Hyr1p on *C. albicans*

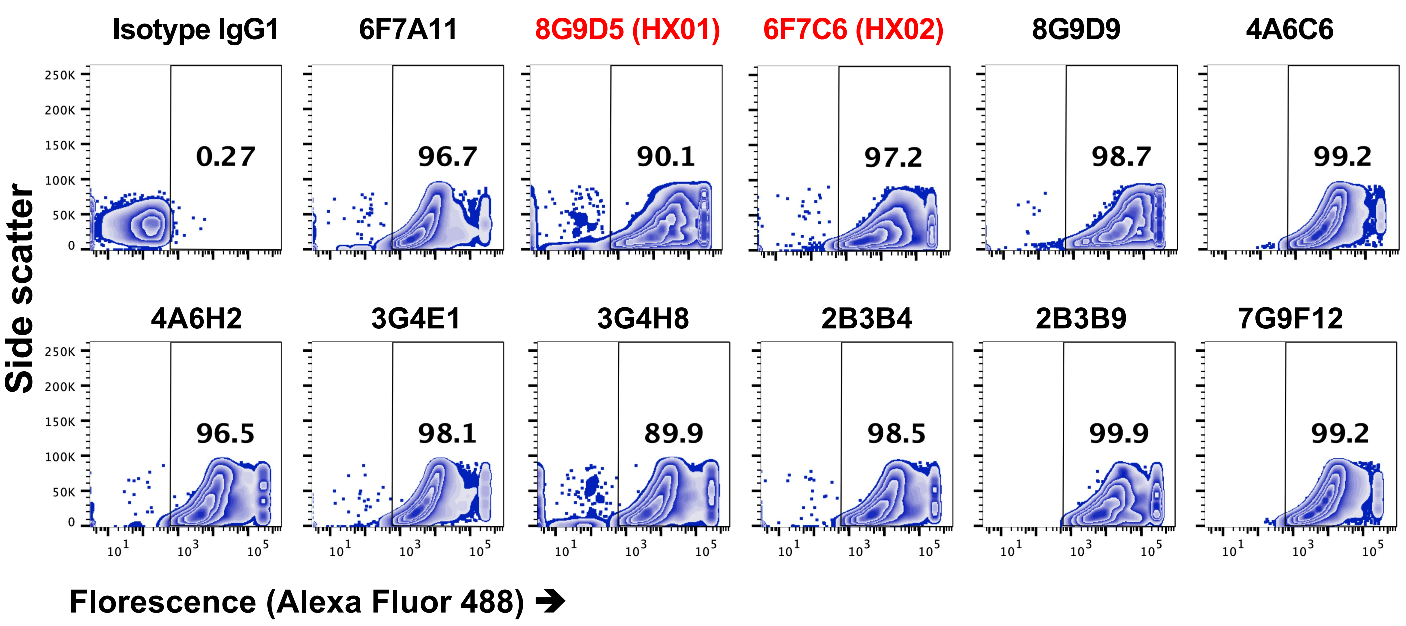

Figure S1. Mouse hybridoma clones produced monoclonal antibodies that recognize Cal-Hyr1#peptide5 and native epitopes on *C. albicans*. **A.** Culture supernatants obtained from hybridoma cells producing anti-Hyr1 peptide#5 MAbs bind Hyr1 peptide#5 in ELISA. Data are presented as OD450 values. **B.** 2 x 10^6 cells of germinated *C. albicans* were incubated with culture supernatants obtained from hybridoma cells producing anti-Hyr1 peptide#5 MAbs or isotype-matched control IgG. Bound MAb were detected by anti-mouse IgG1 labelled with AlexaFluor 488. The extent of binding was quantified by flow cytometry. Clone 8G9D5 (HX01) and 6F7C6 (HX02) were chosen for monoclonal antibody production. Data are presented in a zebra plot highlighting the percentage of each organism that were bound by each Ab.

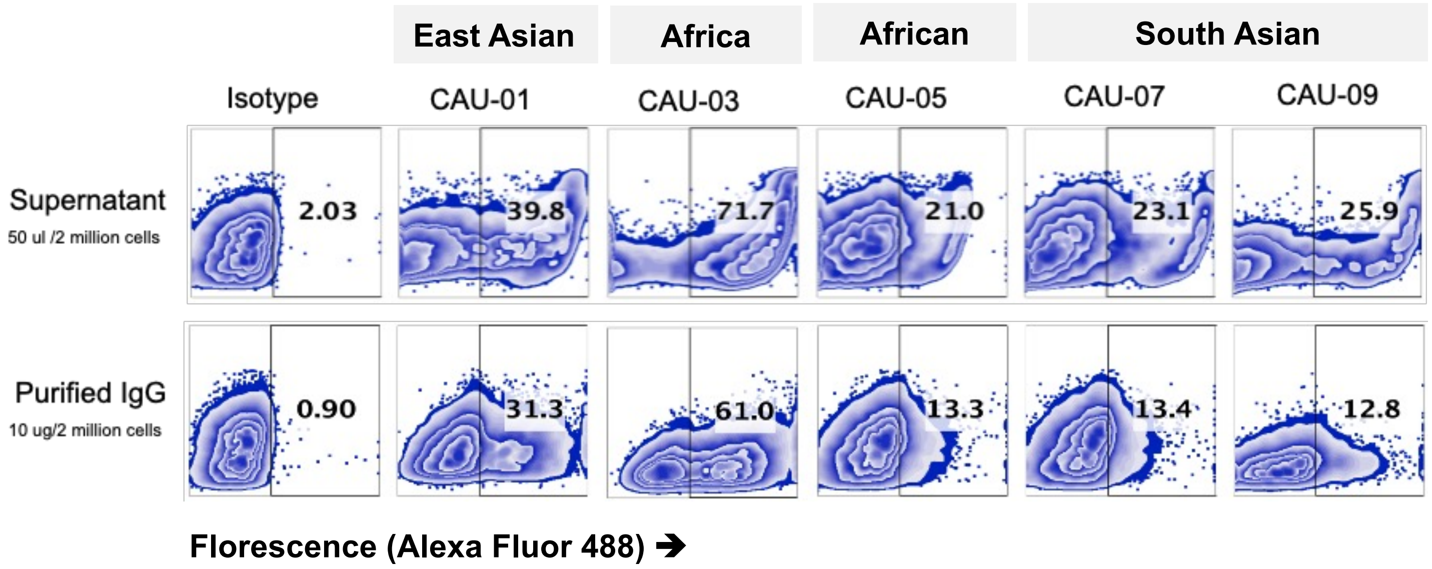

Figure S2. Anti-Cal-Hyr1 mAb (HX01) binds to different *C. auris* isolates. 2 x 10^6 cells of each organism *C. auris* isolates (representing all four clades) were incubated with 10 µg/ml of HX01 MAb or isotype-matched control IgG1. Bound MAb were detected by anti-mouse IgG1 labelled with AlexaFluor 488. The extent of binding was quantified by flow cytometry. Data are presented in a zebra plot highlighting the percentage of each organism that were bound by each Ab.

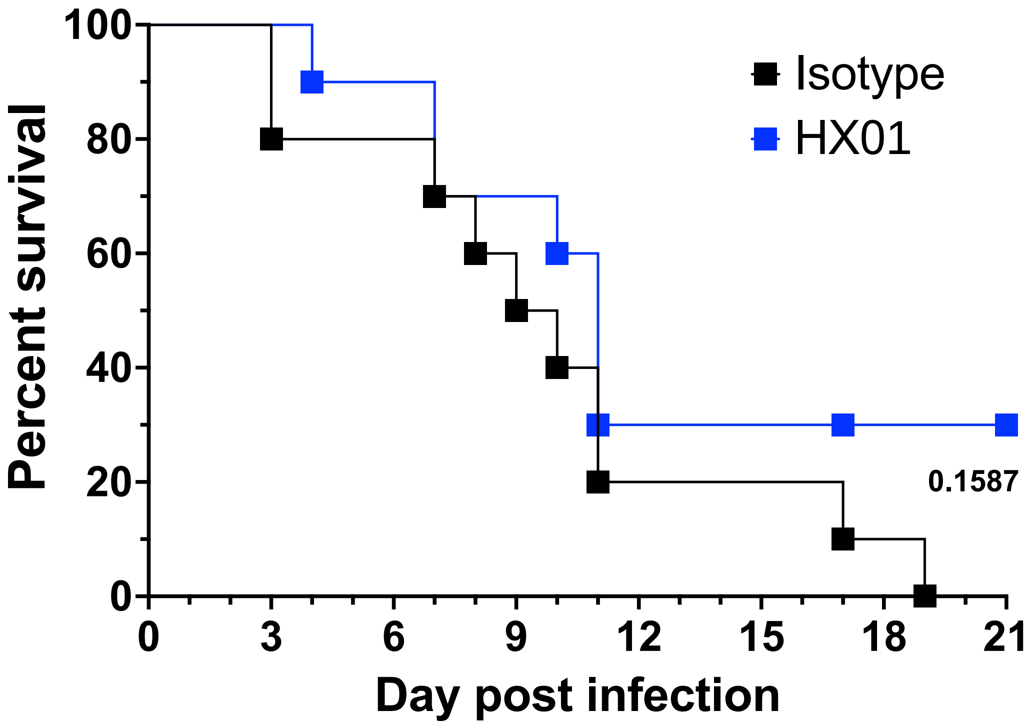

Figure S3. Anti-Cal-Hyr1 mAb (HX01) does not protect against systemic *C. albicans infection*. Immunocompetent 4-6 weeks old ICR CD-1 mice (n=10 /group) were infected with 2 x 10^5 cells/mouse through tail-vein injections. Mice were treated with either 30 ug/mouse of HX01 or isotype control antibody through intraperitoneal injections on day +1. and +8 post infection. Mice survival was compared by Log-rank Test, The p values <0.05 was considered significant.
